# Prognostic Factors for Recurrence in Esophageal Cancer Patients Treated With Neoadjuvant Therapy and Surgery: A Single-institution Analysis

**DOI:** 10.7759/cureus.8108

**Published:** 2020-05-14

**Authors:** Misbah Khan, Namra Urooj, Aamir Ali Syed, Shahid Khattak, Ather Kazmi, Mohammad I Ashraf, Sadaf Batool

**Affiliations:** 1 Surgery, Shaukat Khanum Memorial Cancer Hospital and Research Centre, Lahore, PAK; 2 Surgical Oncology, Shaukat Khanum Memorial Cancer Hospital and Research Centre, Lahore, PAK; 3 Radation Oncology, King Faisal Specialist Hospital and Research Centre, Riyadh, SAU

**Keywords:** risk factors, recurrence, neoplasm recurrence, disease-free survival, esophageal cancer, prognosis, recurrence local, recurrence locoregional

## Abstract

Background

The purpose of this study is to analyze potential predisposing factors for a higher risk of recurrence in our esophageal cancer patients managed with neoadjuvant chemotherapy, radiation therapy, and surgery, and to determine their impact on disease-free survival (DFS) and time to recurrence.

Methods

A total of 154 of 232 patients staged T1b to T4a managed electively at our institute from July 2005 through July 2015 with a tri-modality approach were retrospectively evaluated. Basic demographic, clinical, radiological, operative, and pathological disease-related parameters, along with waiting time for surgery and type of neoadjuvant modality used, were assessed as potential risk factors. The primary endpoint was the impact of these on the risk of recurrence. The secondary endpoint was to study their relation on DFS and time to recurrence.

Results

The recurrence rate in this particular study was 33.1% over a median follow-up of 35 months (interquartile range = 19-50.3). The median time to recurrence was 12 months, and 94% of recurrences occurred within two years. The median DFS was 33 months, and the one- and three-year DFS was 90% and 72%, respectively. On univariate and multivariate analysis, a complete pathological response (hazard ratio [HR]: 3.8, 95% confidence interval [CI]: 1.41-10.11), negative resection margins (HR: 5.9, 95% CI: 1.69-20.45), and a low nodal index (HR: 6.3, 95% CI: 1.37-28.67 for an index of 0.1-0.2; and HR: 15.2, 95% CI: 0.96-241.73 for an index of >0.2) were found as statistically significant (P = < 0.05) for risk to recurrence. In addition to these three, a low comorbidity index (P = 0.03; HR: 3.5; 95% CI: 1.16-10.52) was an individual positive predictor of DFS.

Conclusions

A complete pathological response, low nodal index, and margin-negative resection were the identified predictors of freedom from recurrence, with a better DFS and a low comorbidity index as additional indicators of prolonged DFS.

## Introduction

Esophageal cancer is a complex disease associated with a very high recurrence rate. It is the sixth most common cause of cancer-related death worldwide [1]. Combined modality treatment with neoadjuvant chemotherapy and radiation therapy (XRt) followed by surgical resection is the current standard of care associated with a better survival and disease control as compared with either treatment modality alone [2,3]. The reported overall five-year survival after perioperative chemotherapy is 38%, with a disease-free survival (DFS) of 34 % at five years [4]. On the contrary, a three-year overall survival rate of 50% after esophagectomy along with neoadjuvant chemoradiation therapy and a locoregional control rate reaching up to 80% at three years have been reported [5-8]. Nevertheless, the reported recurrence rate after the radical tri-modality treatment strategy is still up to 35% to 42% [3,9].

Our hospital is a high-volume center for esophageal cancer management. As presented in our adult patient population, esophageal cancer is the sixth most common overall and the fifth most common cancer among solid organ malignancies seen at our center [10]. Standard management protocol for resectable esophageal cancer T1b and above is neoadjuvant chemotherapy and XRt followed by surgical resection and en bloc lymph node dissection. However, it is not infrequent to see recurrent disease in these patients after treatment completion. The data from our center have shown a recurrence rate of 39% in this treated set of the population [11]. In the absence of any measurable tools or pre-established defining factors to predict the occurrence of treatment failure, it is imperative to study the patient populations who develop a poor outcome [12]. A study in this population could pave the way in segregating patient groups at a higher risk of recurrence, allowing the development of enhanced surveillance programs for such identified clusters with a final goal of defining strategies at its prevention.

The goal of this study was to analyze potential predisposing factors for a higher risk of recurrence in our esophageal cancer patients managed with neoadjuvant chemotherapy, XRt, and surgery, and to determine their impact on DFS and time to recurrence.

## Materials and methods

An overview of this study was initially presented as an abstract at the 16th Shaukat Khanum Cancer Symposium, 2017, and at the National Comprehensive Cancer Network Annual Conference, 2019 (unpublished abstract: Misbah Khan, Prognostic Factors for Recurrence in Esophageal Cancer Patients Treated with Neoadjuvant Therapy and Surgery, NCCN 2019 Annual Conference, Improving the Quality, Effectiveness and Efficiency of Cancer Care, March 22, 2019) [13]. The full details of the study are presented herein. Our study was a retrospective analytic design. All potentially resectable esophageal cancer patients at our institute from July 2005 through July 2015 with an elective multidisciplinary management plan of neoadjuvant treatment followed by esophageal resection were evaluated. After a formal exemption status for the study was granted by the Institutional Review Board, basic demographics, clinical, radiological, and pathological disease-related parameters were assessed through the hospital information system for a total of 232 patients.

Patients with an emergency esophagectomy (n=8) and hence no neoadjuvant treatment, histopathology indicating something other than adenocarcinoma or squamous cell cancer, and a Siewert type 3 tumor location on initial endoscopy were excluded. Moreover, patients with metastatic disease at presentation and before completion of treatment were considered ineligible. For documentation of recurrence, patients with a less than one year of follow-up period, including those with early mortality due to causes other than a recurrent disease, were excluded. The last date of follow-up for all the patients was June 8, 2018. Of 187 patients, 15 died earlier than six months, and 18 were lost to follow-up (unable to complete a minimum one-year follow-up) and were omitted from the final data.

The variables explored for a relationship with recurrence in addition to the basic patient-related demographic variables were tumor histological type, pathological grade, location, initial radiological TNM (tumor, node, metastasis) stage, pathological nodal index, completeness of margin status, type of neoadjuvant modality used, type of surgical procedure performed, post-operative course (major class III/IV complications and extended hospital stay), and duration between the completion of neoadjuvant treatment and surgical procedure performed (Table 1).

**Table 1:** Patient demographic and tumor characteristics Co-morbidity classification as per the Charlson Comorbidity Index. IIClavien-Dindo Classification system for post-operative complications. FU, fluorouracil; CR, complete pathological response; GOJ, gastroesophageal junction; XRt, radiation therapy; body mass index

Variable	Total	Recurrence	No Recurrence
	N (%), 154 (100)	N (%), 51 (33.1)	N (%), 103 (66.9)
Gender			
Men	79 (51.3)	28 (54.9)	51 (49.5)
Women	75 (48.7)	23 (45.1)	52 (50.5)
Comorbidity index^I^			
0-1	139 (90.3)	43 (84.3)	96 (93.2)
>1	15 (9.7)	8 (15.7)	7 (6.8)
Histological subtype			
Adenocarcinoma	35 (22.7)	15 (29.4)	20 (19.4)
Squamous cell carcinoma	119 (77.3)	36 (70.6)	83 (80.6)
Pathological tumor grade			
Well-differentiated	17 (11)	3 (5.9)	14 (13.6)
Moderately	100 (64.9)	36 (70.6)	64 (62.1)
Poorly	34 (22.1)	12 (23.5)	22 (21.4)
Tumor initial radiological stage			
Early (stage I-IIb)	36 (23.4)	9 (17.6)	27 (26.2)
Late (stage IIIa and above)	118 (76.6)	42 (82.4)	76 (73.8)
Tumor location			
Mid esophageal	104 (67.5)	36 (70.6)	68 (66.0)
Distal/GOJ Siewert type 1	40 (26)	8 (15.7)	32 (31.1)
GOJ Siewert type 2	10 (6.5)	7 (13.7)	3 (2.9)
Neoadjuvant type			
Carboplatin-paclitaxel based XRt	53 (34.4)	16 (31.4)	37 (35.9)
5-FU based XRt	81 (52.6)	23 (45.1)	58 (56.3)
Magic protocol	15 (9.7)	10 (19.6)	5 (4.9)
Incomplete	5 (3.2)	2 (3.9)	3 (2.9)
Pathological response to neoadjuvant			
CR	85 (55.2)	17 (33.3)	68 (66.0)
Without CR	69 (44.8)	34 (66.7)	35 (34.0)
Type of surgery			
Conventional	63 (40.9)	25 (49.0)	38 (36.9)
Minimally invasive	65 (42.2)	21 (41.2)	44 (42.7)
Hybrid	26 (16.9)	5 (9.8)	21 (20.4)
Oncological resection margin			
Negative	122 (79.2)	29 (56.9)	93 (90.3)
Positive	32 (20.8)	22 (43.1)	10 (9.7)
Post-operative/major complications class III/IV^II^			
No	130 (8.4)	42 (82.4)	88 (85.4)
Yes	24 (15.6)	9 (17.6)	15 (14.6)
Hospital stay			
<11 days	118 (76.6)	39 (76.5)	79 (76.7)
>11 days	36 (23.4)	12 (23.5)	24 (23.3)
Nodal index			
<0.1	129 (83.8)	32 (62.7)	97 (94.2)
0.1-0.2	15 (9.7)	11 (21.6)	4 (3.9)
>0.2	10 (6.5)	8 (15.7)	2 (1.9)
Interval between neoadjuvant and surgery			
Standard	57 (37.0)	18 (35.2)	39 (37.9)
Delayed	94 (61.0)	31 (60.8)	63 (61.2)
Mean + standard error of the mean			
Age(years)	52.6 + 0.86	54.7 + 1.46	51.6 + 1.06
<55 years	83 (53.9)	25 (49.0)	58 (56.3)
>55 years	71 (46.1)	26 (51.0)	45 (43.7)
BMI (kg/m^2^)	22.4 + 0.35	21.9 +0.58	22.5 + 0.43
18-25	98 (63.6)	29 (56.8)	69 (67)
>25	36 (23.4)	11 (21.6)	25 (24.3)
<18	20 (13)	11 (21.6)	9 (8.7)
Mean number of lymph nodes harvested	13.5 + 0.44	12.2 + 0.72	14.1 + 0.54
Length of follow-up (in months)	36.8+ 1.63	19.8+ 1.53	45.5+ 1.81

For an optimal oncological and surgical quality assessment purpose, we have included variables of margin status, a pathological response to neoadjuvant treatment, and nodal index instead of pathological T and N stage. Table 2 shows the detailed pre- and post-neoadjuvant stage distribution and the extent of down-staging achieved in our study group.

**Table 2:** Detailed stage distribution pre and post-neoadjuvant treatment

	Total	Recurrence	No Recurrence
	N (%), 154 (100)	N (%), 51 (33.1)	N (%), 103 (66.9)
Tumor initial T stage			
T1a, T2	10 (6.5)	2 (3.9)	8 (7.8)
T3	107 (69.5)	33 (64.7)	74 (71.8)
T4b	37 (24)	16 (31.4)	21 (20.4)
Tumor initial N stage			
N0	37 (24)	12 (23.5)	25 (24.3)
N1, N2	117 (76)	39 (76.5)	78 (75.7)
Pathological T stage			
T0	85 (55.2)	17 (33.3)	68 (66.0)
T1-3	67 (43.5)	32 (62.8)	35 (34.0)
T4	2 (1.3)	2 (3.9)	0 (0.0)
Pathological N stage			
N0	125 (81.2)	30 (58.8)	95 (92.2)
N1	18 (11.7)	12 (23.5)	6 (5.8)
N2	8 (5.2)	6 (11.8)	2 (1.9)
N3	3 (1.9)	3 (5.9)	0 (0.0)

We did not include advanced stage tumors T4b or M1 in our study. Hence, for analysis, the initial radiological stage was divided into early stages 1 and 2 and late-stage 3A and above [14]. A circumferential margin of less than 1 mm and a longitudinal margin of <1 cm on the final histopathology report was defined as positive or close. Waiting time of more than 6 weeks after neoadjuvant chemotherapy and more than 12 weeks after chemoradiation was considered as delayed.

The primary endpoint was to evaluate the impact of these prognostic factors on the risk of recurrence in the group, which developed recurrence, as compared with the patient group who remained recurrence-free. The secondary endpoint is to study the relation of the aforementioned factors on DFS and time to recurrence.

For all middle to lower esophageal cancers, staged T1b and above, standard management protocol at our center is neoadjuvant induction chemotherapy (5-fluorouracil [FU]/platinum-based or newer carboplatin-paclitaxel based regimen) followed by concurrent chemoradiation therapy (45 Gy in 25 fractions), with an assessment and proceed to surgery at six to eight weeks. Some cases of adenocarcinoma of the true cardia (Siewert type 2) on final histopathological assessment also received perioperative chemotherapy on MAGIC protocol as per multidisciplinary board decision based on their initial diagnostic assessment (endoscopic ultrasound and contrast-enhanced computed tomography [CT]). Additionally, a positron emission tomography (PET)-CT in all patients and a staging laparoscopy for Siewert type 2 adenocarcinoma was performed to rule out metastatic elements before the start of a curative intent treatment protocol.

Patients were monitored through follow-up at regular intervals after completion of treatment with both surgery and oncology clinics. The standard guideline for follow-up was the first visit at two weeks after surgery to review and discuss the histopathology report followed by visits at one and three months. Then patients were monitored through follow-up at six-month intervals for the initial two years post-surgery and yearly thereafter for the next three years. A CT of the chest and abdomen with contrast was repeated at a one-year interval unless prompted for by symptoms or signs of recurrence. Endoscopy was only performed for the evaluation of any significant dysphagia associated symptoms. If recurrence was suspected by the above investigations, additional confirmatory tests including PET-CT and biopsy are advised.

Statistical analysis

It was a retrospective analytic study design. All data were analyzed using SPSS Statistics for Windows, Version 20.0. (IBM Corp., Armonk, NY, USA). Pearson’s chi-square test for categorical variables and one-way analysis of variance for continuous variables, along with binary logistic regression analysis, were used for establishing univariate and multivariate associations of all prognostic factors for the assessment of risk to recurrence. Cox regression analysis was used to assess time to recurrence and DFS and the impact of prognostic factors on these two variables. For regression analysis, continuous variables were dichotomized according to their mean values or split into sub-groups depending on their clinical significance. For all practical purposes, a P-value of 0.05 or less was considered significant.

## Results

A total of 154 patients with a median follow-up of 35 months (interquartile range [IQR] = 19-50.3 months) were further evaluated. The median age of our selected patient population was 53.5 years (IQR = 47-60 years), and their body mass index (BMI) was 21.7 (IQR = 19.6- 25.1). The median length of hospital stay was nine days (IQR = 8-11 days), waiting time for surgery from the date of completion of neoadjuvant treatment was 13 weeks (IQR = 11-18 weeks), and we had a median nodal harvest of 13 (IQR = 10-17).

The length of follow-up was considerably longer for the group without recurrence (median: 43.5 months; IQR = 32-59.3) as compared with the patients who develop a recurrence (median: 16 months; IQR = 12-25.8).

The recurrence rate in this particular study is 33.1%. Depending on patients’ initial mode of presentation, 60.8% were distant metastatic recurrences (31 cases), including peritoneal disease and non-regional lymph nodes. While 21.6% (11 cases) were found to have a locoregional disease relapse, the remaining 17.6% (nine cases) had both locoregional and metastatic recurrent disease.

**Figure 1:**
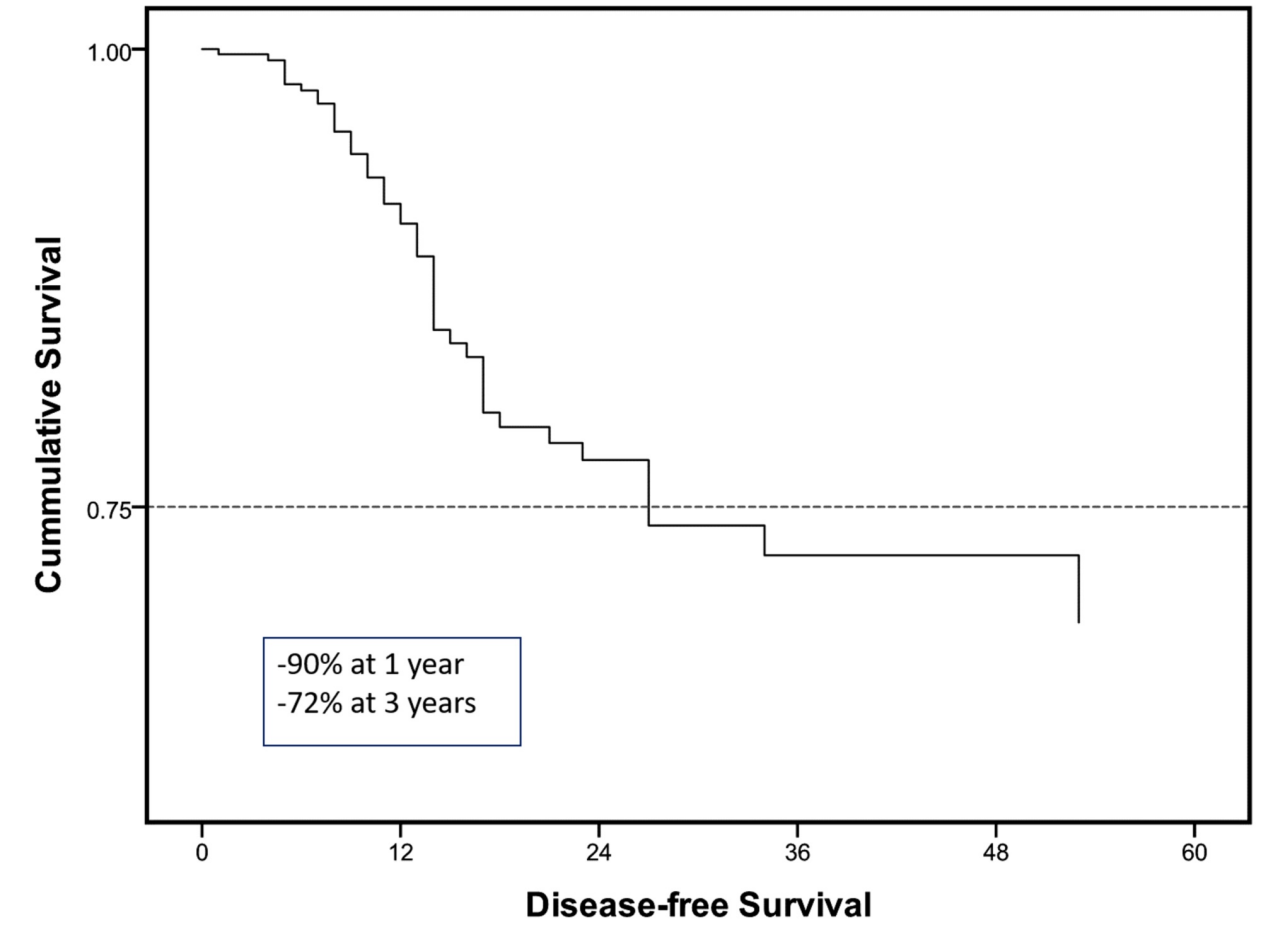
Cox regression analysis for overall disease-free survival at the mean of covariates

On univariate and multivariate linear logistic regression analysis after controlling for all included variables in the study, a complete pathological response (CR), negative resection margins, and a low nodal index were found as statistically significant (P ≤ 0.05) predictors of freedom from recurrence (Table 3). Overall DFS on Cox regression survival plots showed similar trends (Figure 1), with a median DFS of 33 months and a one- and three-year DFS of 90% and 72%, respectively.

**Table 3:** Univariate and multivariate regression analysis for risk of recurrence CI, confidence interval; BMI, body mass index; SCCA, squamous cell carcinoma; XRt, radiation therapy; FU, fluorouracil

Potential Prognostic Factors	Univariate Analysis			Multivariate Analysis		
	Odds Ratio	P-value	95% CI	Adjusted Odds Ratio	P-value	95% CI
Age < 55 years vs.						
> 55 years	1.34	0.393	0.68-2.63	1.33	0.584	0.48-3.65
Gender: men vs.						
Women	0.81	0.529	0.41-1.58	0.66	0.005	1.69-20.45
BMI: 18-25 vs.						
>25	1.05	0.914	0.46-2.40	1.17	0.804	0.33-4.18
<18	2.64	0.057	0.97-7.18	2.38	0.257	0.53-10.6
Comorbidity index: 0-1 vs.						
>1	2.55	0.08	0.87-7.49	4.13	0.108	0.73-23.24
Histological subtype: SCCA vs.						
Adenocarcinoma	1.82	0.13	0.83-3.98	0.65	0.636	0.11-3.93
Tumor grade: well differentiated vs.						
Moderately differentiated	2.63	0.149	0.71-9.75	1.08	0.931	0.21-5.54
Poorly differentiated	2.55	0.201	0.61-10.65	0.85	0.862	0.14-5.29
Tumor initial radiological stage: Early vs.						
Late (IIIa and above)	1.66	0.237	0.71-3.85	3.06	0.078	0.88-10.62
Tumor location: mid-esophageal vs.						
Siewert type 1	0.47	0.092	0.19-1.13	0.17	0.03	0.04-0.84
Siewert type 2	4.41	0.039	1.07-18.08	0.83	0.904	0.04-17.74
Neoadjuvant type: carboplatin-paclitaxol based XRt vs.						
5-FU based XRt	0.92	0.823	0.43-1.96	0.85	0.762	0.29-2.50
Magic protocol	4.63	0.014	1.36-15.72	1.97	0.56	0.20-19.34
Incomplete	1.54	0.652	0.24-10.13	0.57	0.73	0.02-13.64
Type of surgery: conventional vs.						
Minimally invasive	0.73	0.385	0.35-1.49	0.81	0.75	0.22-2.94
Hybrid	0.36	0.07	0.12-1.09	0.35	0.204	0.07-1.77
Waiting time for surgery: standard vs.						
Delayed	1.07	0.859	0.53-2.16	2.13	0.164	0.73-6.17
Length of hospital stay: <11 days vs.						
>11 days	1.01	0.975	0.46-2.24	1.23	0.738	0.36-4.18
Post-operative: no vs.						
Major complications	1.29	0.583	0.52-3.19	1.27	0.74	0.32-5.07
Complete pathological response vs.						
Partial or no	3.89	0	1.91-7.91	3.78	0.008	1.41-10.11
Oncological complete resection vs.						
Margin-positive	7.06	0	2.99-16.60	5.88	0.005	1.69-20.45
Nodal index < 0.1 vs.						
0.1-0.2	8.34	0.001	2.48-28.01	6.26	0.018	1.37-28.67
>0.2	12.13	0.002	2.45-60.07	15.17	0.054	0.96-241.73

**Figure 2:**
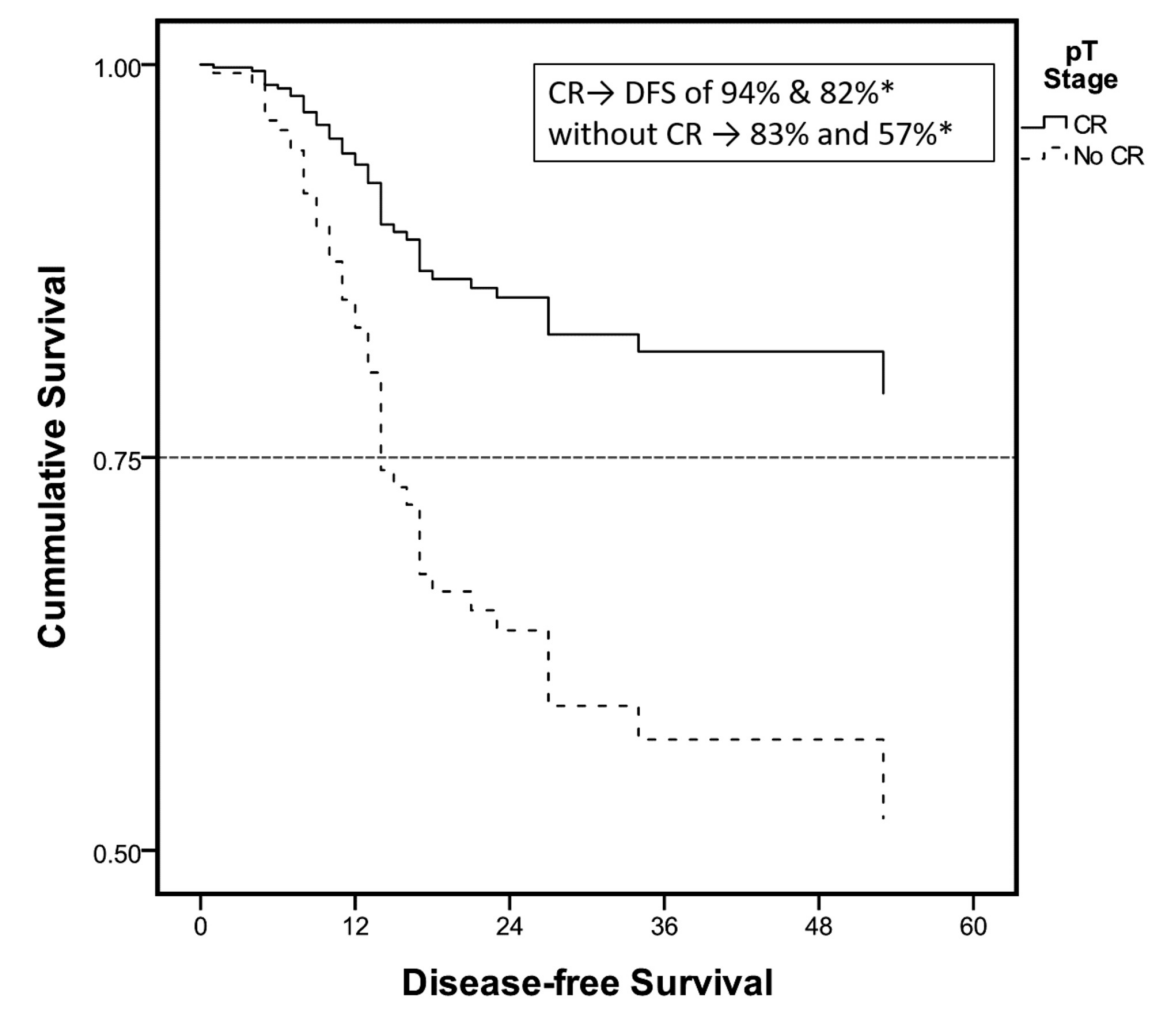
Cox regression survival curves: subgroup analysis Survival curve: pathological stage. *DFS at one and three years. CR, Cox regression; DFS, disease-free survival.

**Figure 3:**
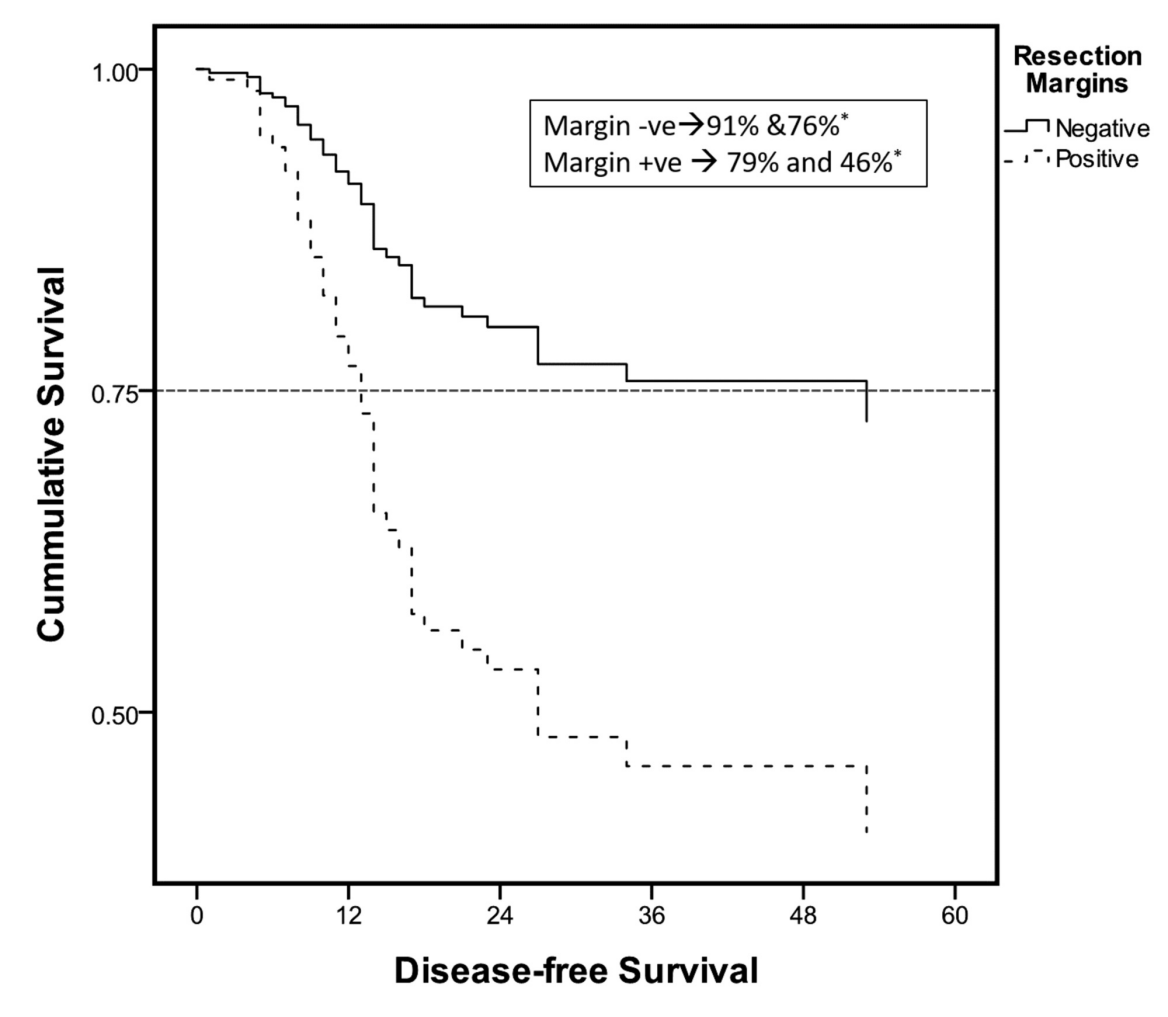
Cox regression survival curves: subgroup analysis Survival curve: resection margin status. *Disease-free survival at one and three years.

**Figure 4:**
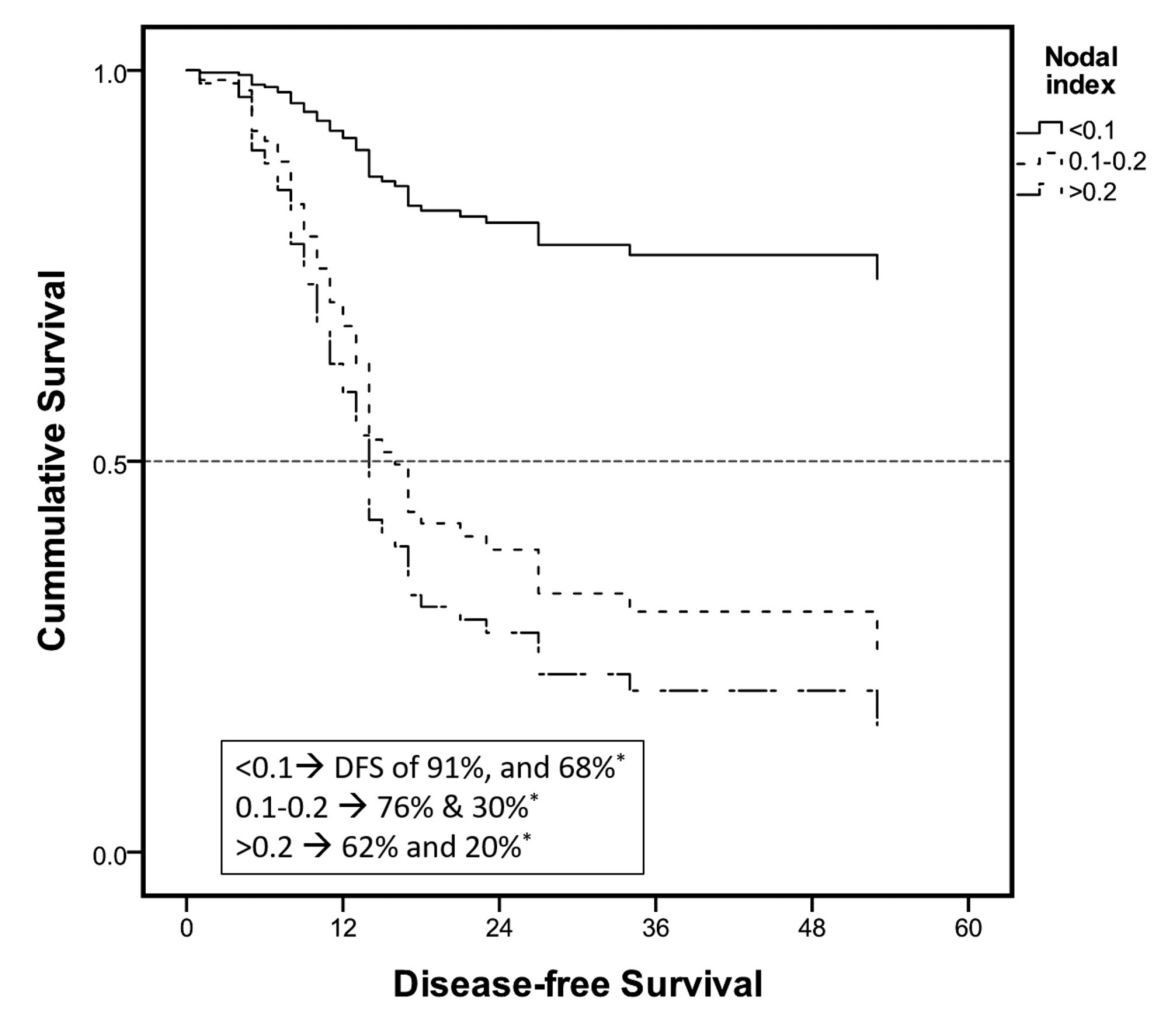
Cox regression survival curves: subgroup analysis Survival curve: nodal index shows the relative degree of impact on DSF of significant prognostic factors. *DFS at one and three years DFS, disease-free survival

**Figure 5:**
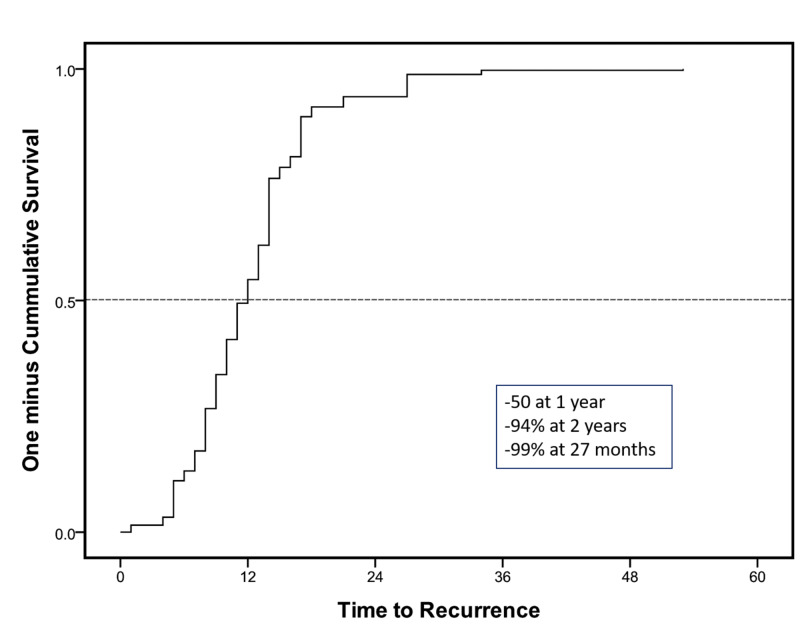
Cox regression survival functions for time to recurrence at the mean of covariates

In addition to the three positive prognostic factors for a risk to recurrence, a low comorbidity index was also an individual predictor of DFS (Figures 2-4). The median time to recurrence was 12 months (Table 4). Most of the recurrences occurred within the first two years (94%) and 99% within 27 months after surgery (Figure 5). Only a couple of cases were reported late at 34 and 53 months; one was adenocarcinoma and the other squamous, managed with magic protocol and 5-FU based therapy, respectively. However, on the Cox regression model, none of the individual factors had any statistically significant impact on time to recurrence (Table 5).

**Table 4:** Cox survival statistics SE, standard error; IQR, interquartile range

Survival Duration (in Months)	Mean + SE		
	Median (IQR)		
	Total	Recurrence	No Recurrence
Overall disease-free survival	34.9+ 1.75	14 + 1.29	45.5 + 1.81
	33 (14-50.3)	12.5 (8-17)	43.5 (32-59.3)
Time to recurrence	13.3 + 1.25		
	12 (8-16.3)		

**Table 5:** Cox regression analysis for disease-free survival and time to recurrence CI, confidence interval; BMI, body mass index; SCCA, squamous cell carcinoma; XRt, radiation therapy

Potential Prognostic Factors	Disease-Free Survival			Time to Recurrence		
	Hazard ratio	P-value	95% CI	Hazard Ratio	P-value	95% CI
Age < 55 years vs.											
>55 years	1.21	0.608	0.59-2.45	0.83	0.718	0.30-2.28
Gender: men vs.						
Women	0.83	0.62	0.39-1.76	1.95	0.263	0.61-6.31
BMI: 18-25 vs.						
>25	1	0.993	0.41-2.47	0.68	0.569	0.19-2.53
<18	1.45	0.408	0.60-3.50	0.8	0.732	0.22-2.87
Comorbidity index 0-1 vs.						
>1	3.5	0.026	1.16-10.52	1.01	0.987	0.21-4.86
Histological subtype: SCCA vs.						
Adenocarcinoma	1.35	0.627	0.41-4.47	3.8	0.136	0.66-22.00
Tumor grade well differentiated vs:						
Moderately differentiated	1.19	0.796	0.32-4.51	0.38	0.317	0.06-2.51
Poorly differentiated	0.8	0.767	0.18-3.54	0.97	0.975	0.17-5.55
Tumor initial radiological stage: early vs.						
Late (IIIa and above)	2.05	0.098	0.88-4.80	1.84	0.326	0.54-6.25
Tumor location: Mid-esophageal vs.						
Siewert type 1	0.2	0.006	0.007-0.63	0.94	0.942	0.16-5.61
Siewert type 2	0.47	0.385	0.09-2.57	0.24	0.218	0.03-2.31
Neoadjuvant type: Carboplatin-paclitaxel based XRt vs.						
5-FU based XRt	0.6	0.237	0.26-1.39	0.7	0.545	0.22-2.53
Magic protocol	0.53	0.431	0.11-2.56	0.13	0.028	0.02-0.80
Incomplete	0.74	0.791	0.08-7.12	0.68	0.774	0.05-9.20
Type of surgery conventional vs.						
Minimally invasive	1.02	0.972	0.43-2.38	0.95	0.933	0.29-3.16
Hybrid	0.6	0.4	0.18-1.98	1.32	0.781	0.18-9.50
Waiting time for surgery: standard vs.						
Delayed	1.28	0.47	0.66-2.50	0.57	0.307	0.19-1.69
Length of hospital stay < 11 days vs.						
>11 days	1.13	0.775	0.48-2.68	0.83	0.783	0.22-3.13
Post-operative: no vs.						
Major complications	1.49	0.432	0.55-3.99	1.23	0.793	0.27-5.61
Complete pathological response vs.						
Partial	2.78	0.006	1.33-5.80	2.45	0.099	0.84-7.09
Oncological complete resection vs.						
Margin positive	3.61	0.004	1.50-8.68	1.99	0.21	0.68-5.81
Nodal index < 0.1 vs.						
0.1-0.2	4.35	0.002	1.72-11.04	1.9	0.285	0.59-6.16
>0.2	5.71	0.006	1.65-19.73	4.04	0.071	0.89-18.34

## Discussion

Our data suggest that in post-esophagectomy patients who are treated in combination with neoadjuvant treatment modalities, the only significant predictors for a higher risk to recurrence are higher nodal index, a positive resection margin, and residual disease. An additional risk factor for a poor DFS was a greater comorbidity index score.

A number of studies have analyzed factors influencing recurrence in patients with esophageal cancer post-surgical treatment with or without neoadjuvant chemoradiation therapy. In our series, the rate of recurrence was 33.1%, which was comparable to the reported recurrence rate of other studies (42.4% and 59.2%) [9,15]. Lee et al. in their series of 465 resectable esophageal cancer patients identified the following variables as independent predictors of recurrence: performance status greater than 0, poor tumor differentiation, en bloc resection, and advanced pathological stages (II/III/IV), on a multivariate regression model adjusted for P stage. Although they also found induction therapy to be an independent factor, unlike our patient population, more than 50% of their patients did not receive any neoadjuvant treatment [9]. In another similar study of 1,002 consecutive squamous cell carcinoma patients, Xu et al. concluded that the variables associated with a higher rate of recurrence by multivariate analyses were gender (hazard ratio [HR]: 1.7; 95% confidence interval [CI]: 1.2 2.5; P = 0.002), depth of invasion (HR: 1.4; 95% CI: 1.2 1.6; P < 0.001), and lymph node involvement (HR: 1.4; 95% CI: 1.3 1.5; P < 0.001) [15]. They excluded patients who received neoadjuvant treatment, and the patient population was considerably non-homogenous based on their adjuvant protocols. Our study failed to establish any association with the performance status and degree of tumor differentiation or gender with the rate and time of recurrence.

In our series, the median DFS was 33 months. While one- and three-year DFS was 90% and 72%, respectively, it was a predicting factor that was statistically associated with a higher DFS and included CR, negative resection margins, a low nodal index, and a low comorbidity index. Lee et al. showed independent predictors of DFS to be the same as predictors of risk to recurrence in their series except for the histopathological tumor grade [9]. Eng et al. analyzed factors associated with overall survival after a tri-modality treatment in esophageal adenocarcinoma. On Cox regression analysis, characteristics associated with a decreased overall survival included tumor stage, lymphovascular invasion, positive surgical margins, age, and comorbidity index. In addition, their results have demonstrated that post-operative adjuvant chemotherapy is also associated with increased survival in node-positive sub-group [12]. Another study by Yuequan et al. examined 1,553 cases of esophageal squamous cell carcinoma with a heterogeneous population of approximately 50% receiving adjuvant chemotherapy or XRt and the rest receiving upfront surgery. In this particular series, with an overall survival of 43.7% at three years, the independent prognostic factors they identified in addition to the stage of the cancer were the degree of tumor differentiation and family history of esophageal cancer [16].

The subject of optimal lymph node harvest has been a matter of constant debate. When the total number of resected nodes was examined as a categorical variable by Altorki et al. in their series of 264 patients without neoadjuvant therapy, for node-positive patients, the death hazard was significantly reduced for those with a higher total number of lymph nodes examined (P < 0.05). While for node-negative patients, a significantly reduced hazard was present only when more than 40 nodes were resected (HR = 0.23; P = 0.01) [17]. In this particular study, for multivariate and Cox regression analysis, the nodal index was used instead as a more relevant measure of recurrence [5,18]. Conversely, a more recent sub-group analysis of the Shapiro et al. failed to show any association of the total number of lymph nodes harvested with the survival in the patient population who received neoadjuvant chemoradiation therapy prior to surgery [6,19].

Since the management protocol of esophageal cancer as far as standard tri-modality treatment is concerned stays independent of the tumor histological subtype, our study included both adenocarcinoma and squamous cell carcinoma patients. Moreover, for most of the cases, it is the disease stage and the patient’s fitness level to undergo the protracted treatment course that define the treatment strategy. In our study, tumor histological subtype, as well as tumor location and degree of differentiation, failed to show any impact on any of the studied oncological parameters.

The rate of CR in this particular study from our center, keeping into consideration that it did not include patients who progressed on neoadjuvant treatment, was 55.2%. The recurrence rate among the CR group was 20% as compared with the overall recurrence rate among patients with residual disease of 49.3%. The percentage of CR among operated cases of squamous was 63%, and the recurrence rate among this particular group was 26.7, making the rising question of wait-and-watch policy in this particular set of the population more conceivable [6]. CR among adenocarcinoma population was only 26.5%.

In our study, a waiting time of more than 6 weeks after neoadjuvant chemotherapy and more than 12 weeks after chemoradiation was considered as delayed, although a cutoff of eight weeks is usually used in the literature to study perioperative complications. In several studies, a long wait has been linked with a higher CR rate [1-3,20]. This was used to justify a delay in esophagectomy beyond eight weeks for patients who have not yet recovered from chemoradiation [21,22]. In our series, the median time to surgery after completion of neoadjuvant chemoradiation was 13 weeks (IQR = 11-18), and it remains an insignificant variable in terms of time to recurrence and DFS.

The identified risk factors of advanced pathological tumor (T) and nodal (N) stage, along with histopathological differentiation, have been proven significant determinants of recurrence-free survival for other solid organ malignancies [23-25]. This necessitates their further evaluation with more rigorous study protocols in order to assign this high-risk group either to a better surveillance protocol or to offer them additional treatment to decrease the likelihood of recurrence [12,26]. Until then, these results can motivate clinicians to remain critical in their patient selection and evaluate their clinical practice.

## Conclusions

Our data suggest that it is the final histopathology report, elaborating upon the disease response to neoadjuvant treatment in terms of final pathological T and N stage, along with the surgical quality in terms of oncologically complete resection and good lymph node harvest, that determines the prognosis of non-metastatic esophageal cancer patients after a completed tri-modality treatment. Our study concludes that histopathological stage, nodal status, and resection margin status are of utmost importance in determining the recurrence of resectable esophageal cancer in patients who had neoadjuvant chemoradiation therapy.
